# Effect of Culture Conditions on Metabolite Production of *Xylaria* sp.

**DOI:** 10.3390/molecules20057940

**Published:** 2015-04-30

**Authors:** Hongqi Zhang, Zhangshuang Deng, Zhiyong Guo, Yan Peng, Nianyu Huang, Haibo He, Xuan Tu, Kun Zou

**Affiliations:** 1 Hubei Key Laboratory of Natural Products Research and Development, College of Biological and Pharmaceutical Sciences, China Three Gorges University, Yichang 443002, China; E-Mails: zhq6396549@163.com (H.Z.); zhiyongguoctgu@hotmail.com (Z.G.); hny115@126.com (N.H.); haibohe2001@gmail.com (H.H.); tuxuan7@126.com (X.T.); 2 The First College of Clinical Medical Science, China Three Gorges University, Yichang 443002, China; E-Mail: ycpy0303@126.com

**Keywords:** *Xylaria* sp., cryptic natural product, cytochalasin, α-pyrone, xylapyrones, cytotoxicity

## Abstract

Seeking a strategy for triggering the cryptic natural product biosynthesis to yield novel compounds in the plant-associated fungus *Xylaria* sp., the effect of culture conditions on metabolite production was investigated. A shift in the production of five known cytochalasin-type analogues **1**–**5** to six new α-pyrone derivatives, xylapyrones A–F (compounds **6**–**11**), from a solid to a liquid medium was observed. These compounds were identified by analysis of 1D and 2D NMR and HRMS data. Compounds **1**–**3** showed moderate cytotoxicity against HepG2 and Caski cancer cell lines with IC_50_ values ranging from 25 to 63 μM and compounds **4**–**11** were found to be inactive, with IC_50_ values >100 μM.

## 1. Introduction

**Figure 1:**
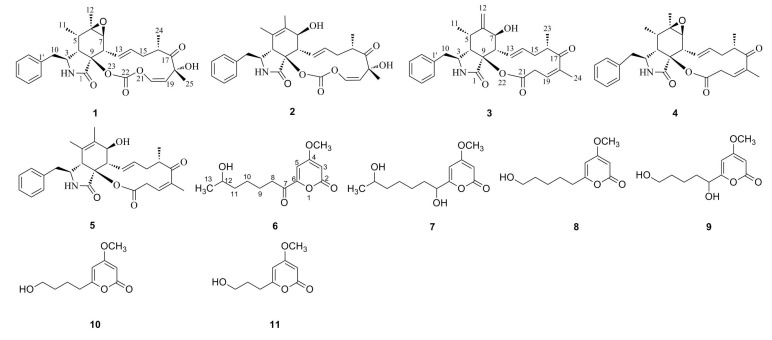
Structures of compounds **1**–**11**.

Natural products from microorganisms are a vital source for innovative therapeutic agents and drug leads [1]. Unfortunately, the high rediscovery rate of known compounds in traditional screening methods has completely frustrated researchers [2]. Secondary metabolism of microbes is regulated by large amounts of genes encoding biosynthetic enzymes and therefore a variety of secondary metabolites should be produced [3,4]. In fact, only a minority of pathway genes is expressed under standardized laboratory conditions and many valuable compounds are overlooked. In order to exploit the full metabolic potential of microorganisms, many regulatory strategies to activate cryptic pathways to facilitate the discovery of new natural products through modification of culture conditions [5], external cues [6], stress [7], co-cultures [8] and genomic approaches [9] were described in the literature. In our approach, by altering easily accessible culture conditions from a solid to a liquid medium, a shift in the production of reported cytochalasin-type analogues **1**–**5** to hitherto unknown α-pyrone derivatives **6**–**11** was observed in the plant-associated fungus *Xylaria* sp. This paper describes the isolation, structure elucidation, and cytotoxic activities of the isolated compounds **1**–**11** (Figure 1). 

## 2. Results and Discussion

### 2.1. Structure Determination

The known compounds cytochalasin E (**1**), cytochalasin K (**2**), cytochalasin Z16 (**3**), rosellichalasin (**4**) and cytochalasin Z17 (**5**) were identified by comparison of the corresponding ^1^H- and ^13^C-NMR data with published results [10,11,12].

**Figure 2:**
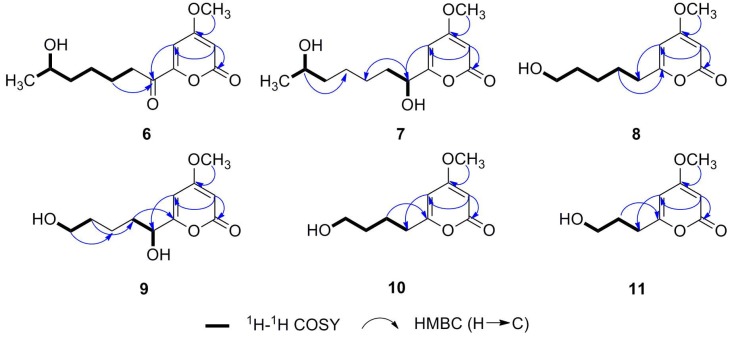
Key HMBC and COSY correlations of compounds **6**–**11** in DMSO.

Compound **6** was obtained as a white amorphous powder. The molecular formula was determined to be C_13_H_18_O_5_ by HREIMS, implying five degrees of unsaturation. Inspection of the ^1^H-NMR spectrum (Table 1 and Supporting Information) reveals signals for two *meta*-coupled aromatic protons at δ_H_ 6.91 and 5.96, three protons at δ_H_ 3.88 (3H, s), one oxygenated methine proton at δ_H_ 3.56, one methylene proton at δ_H_ 2.85, three methylene proton in the higher field at δ_H_ 1.54 (m, 2H), 1.35 (m, 2H) and 1.28 (m, 2H), and one exchangeable proton at δ_H_ 4.32. The ^13^C-NMR and DEPT spectra (Table 1 and Supporting Information) displayed two carbonyl carbons at δ_C_ 193.0 and 161.6, two olefinic methine carbons at δ_C_ 104.6 and 93.5, two sp^2^ quaternary carbon at δ_C_ 169.5 and 153.9, a methoxy carbon at δ_C_ 56.9, an oxygenated methine carbon at δ_C_ 65.6, four methylene carbons at δ_C_ 38.7, 37.2, 24.7 and 23.0, and a methyl carbon at δ_C_ 23.6. The α-pyrone ring was elucidated as 4-methoxy-2-one with a substitutent at C-6 by analysis of HMBC correlations between H_1_-3 (δ_H_ 5.96)/C-2 (δ_C_ 161.6), C-4 (δ_C_ 169.5), C-5 (δ_C_ 104.6), H_1_-5 (δ_H_ 6.91)/C-3 (δ_C_ 93.5), C-4 (δ_C_ 169.5), C-6 (δ_C_ 153.9), and H_3_-OCH_3_ (δ_H_ 3.88)/C-4 (δ_C_ 169.5) (Figure 2). COSY correlations (Figure 2) from H_3_-13 (δ_H_ 1.03), OH-12 (δ_H_ 4.32) via H_1_-12 (δ_H_ 3.56), H_2_-11 (δ_H_ 1.35), H_2_-10 (δ_H_ 1.28) and H_2_-9 (δ_H_ 1.54) to H_2_-8 (δ_H_ 2.85) show the presence of a 2-hexanol group , which is attached to a carbonyl group as indicated by HMBC correlations from H_2_-9 (δ_H_ 1.54) and H_2_-8 (δ_H_ 2.85) to C-7 (δ_C_ 193.0), while H_1_-5 (δ_H_ 6.91) give HMBC correlations (Figure 2) to C-7 (δ_C_ 193.0) implying that C-7 (δ_C_ 193.0) is attached to C-6 (δ_C_ 153.9). The structure of compound **6** was therefore determined to correspond to the previously unreported compound 4-methoxy-6-(6-hydroxy-1-oxoheptyl)-2*H*-pyran-2-one, named xylapyrone A.

**Table 1:** ^1^H- and ^13^C-NMR spectral data (400 MHz, DMSO) of compounds **6**–**8**.

Positionp	6		7		8	
	δ_H_ (mult., *J* in Hz)	δ_C_ (mult.)	δ_H_ (mult., *J* in Hz)	δ_C_ (mult.)	δ_H_ (mult., *J* in Hz)	δ_C_ (mult.)
2		161.6, qC		163.3, qC		164.1, qC
3	5.96, d(2.0)	93.5, CH	5.54, d (2.2)	87.5, CH	5.52, d (2.2)	87.7, CH
4		169.5, qC		171.0, qC		171.5, qC
5	6.91, d(2.0)	104.6, CH	6.10, d (2.2)	97.7, CH	6.05, d (2.2)	99.9, CH
6		153.9, qC		167.8, qC		166.0, qC
7		193.0, qC	4.22, dt (5.0, 7.6)	68.9, CH	2.44, t (7.5)	33.1, CH_2_
8	2.85, t (7.2)	37.2, CH_2_	1.64, m/1.53, m	34.7, CH_2_	1.55, dt (7.5)	26.6, CH_2_
9	1.54, m	23.0, CH_2_	1.30, m	25.1, CH_2_	1.30, m	25.3, CH_2_
10	1.28, m	24.7, CH_2_	1.30, m	24.7, CH_2_	1.42, m	32.6, CH_2_
11	1.35, m	38.7, CH_2_	1.30, m	38.9, CH_2_	3.38, dd (6.4, 11.5)	61.0, CH_2_
12	3.56, m	65.6, CH	3.54, m	65.6, CH		
13	1.03, d (6.1)	23.6, CH_3_	1.01, d (6.2)	23.6, CH_3_		
4-OCH_3_	3.88, s	56.9, CH_3_	3.81, s	56.3, CH_3_	3.79, s	56.7, CH_3_
12-OH	4.32, d (4.9)		4.29, d (5.0)			
7-OH			5.58, d (5.4)			
11-OH					4.36, t (5.1)	

Compound **7** was also obtained as a white amorphous powder and the molecular formula was deduced to be C_13_H_20_O_5_ from the molecular ion peak at *m/z* 256.1308 [M]^+^ (calcd for 256.1311) in the HREIMS, having two more hydrogen atoms than **6**. Detailed NMR spectra (Table 1 and Supporting Information) revealed the existence of the same 4-methoxy-α-pyrone framework as in **6** and that a carbonyl group in **6** has been reduced to a hydroxyl group in **7**. The carbonyl group resonating at δ_C_ 193.0 (C-7) in the NMR spectra of **6** was absent in those of **7**, and instead an oxygenated methine signal at δ_H_ 4.22 (H_1_-7) and δ_C_ 68.9 (C-7) were observed in **7**. These observations suggested the hydroxylation at C-7 and this was verified by the HMBC correlations from H_1_-7 (δ_H_ 4.22) to C-5 (δ_C_ 97.7), C-6 (δ_C_ 167.8), C-8 (δ_C_ 34.7) and C-9 (δ_C_ 25.1) (Figure 2). Thus, the structure of 7 was determined to be as shown in Figure 1, and the compound was named xylapyrone B.

Compound **8** has the molecular formula C_11_H_16_O_4_ suggested by HREIMS data. ^1^H- and ^13^C-NMR data (Table 1 and Supporting Information) showed that **8** had the same basic 4-methoxy-α-pyrone structure with a substituent at C-6, as **6** and **7**. Five methylenes at δ_H_ 3.38, δ_H_ 2.44, δ_H_ 1.55, δ_H_ 1.42 (2H, m) and δ_H_ 1.30 (2H, m) were observed in the ^1^H-NMR spectra. The corresponding carbon signals at δ_C_ 61.0, 33.1, 26.6, 32.6 and 25.3 were present in the ^13^C-NMR spectra. The COSY correlations (Figure 2) of five methylenes suggested the side chain was pentanol. Therefore, the structure of xylapyrone C was unambiguously elucidated as **8**.

Compound **9** was obtained as a white amorphous solid. Its HREIMS exhibits a peak at *m/z* 228.0991 [M]^+^ (calcd for 228.0998), indicating a molecular formula C_11_H_16_O_5_ with one more oxygen atom than **8** [C_11_H_16_O_4_]. The NMR spectroscopic data (Table 2 and Supporting Information) indicated that they were structurally related, and that they differed in their oxidation status. Detailed analysis of the ^1^H- and ^13^C-NMR spectra revealed that the methylene unit at δ_H_ 2.44 and δ_C_ 33.1(CH_2_) of **8** was replaced by an oxygenated methine unit at δ_H_ 4.23 (1H, brs) and δ_C_ 69.5(CH) in **9**. Unexpectedly, no HMBC correlation from H_1_-7 (δ_H_ 4.23) to any other carbon signal was observed, so the location of the hydroxyl group was deduced to be C-7 according to the COSY correlations from OH-7 (δ_H_ 5.60) via H_1_-7 (δ_H_ 4.23) to H_2_-8 (δ_H_ 1.65 and 1.54) and a strong HMBC correlation from H_2_-8 (δ_H_ 1.54) to C-6 (δ_C_ 168.3) (Figure 2). Consequently, the structure of **9** was identified as shown in Figure 1 and named xylapyrone D.

The structures of xylapyrone E (**10**) and xylapyrone F (**11**) were determined straightforwardly as they were very similar to those of **8**. Their NMR data (Table 2 and Supporting Information) and HREIMS suggested the difference of their structures is that side-chains attached to C-6 in **8**, **10** and **11** are pentanol, butanol and propanol, respectively.

**Table 2:** ^1^H- and ^13^C-NMR spectral data (400 MHz, DMSO-*d*_6_) of compounds **9**–**11**.

Position	9		10		11	
	δ_H_ (mult., *J* in Hz)	δ_C_ (mult.)	δ_H_ (mult., *J* in Hz)	δ_C_ (mult.)	δ_H_ (mult., *J* in Hz)	δ_C_ (mult.)
2		163.8, qC		164.2, qC		164.1, qC
3	5.55, d (2.0)	88.1, CH	5.53, d (2.2)	87.7, CH	5.52, d (2.2)	87.7, CH
4		171.5, qC		171.5, qC		171.5, qC
5	6.11, d (2.0)	98.3, CH	6.06, d (2.2)	99.9, CH	6.04, d (2.2)	99.8, CH
6		168.3, qC		166.0, qC		166.0, qC
7	4.23, brs	69.5, CH	2.46, t (7.4)	32.9, CH_2_	2.48, t (7.8)	30.0, CH_2_
8	1.65, m/1.54, m	35.0, CH_2_	1.60, m	23.3, CH_2_	1.70, m	30.0, CH_2_
9	1.34, m	21.7, CH_2_	1.45, m	32.1, CH_2_	3.42, dd (6.0, 11.0)	60.1, CH_2_
10	1.40, m	32.8, CH_2_	3.42, dd (6.0, 11.0)	60.7, CH_2_		
11	3.37, dd (6.0, 11.1)	61.1, CH_2_				
12						
13						
4-OCH_3_	3.81, s	56.8, CH_3_	3.81, s	56.7, CH_3_	3.79, s	56.7, CH_3_
7-OH	5.60, brs					
11-OH	4.35, t (5.0)					
10-OH			4.43, t (5.0)			
9-OH					4.55, t (5.0)	

### 2.2. Cytotoxic Properties

Isolated compounds **1**–**11** were evaluated for *in vitro* cytotoxicity against two cancer cell lines HepG2 and Caski. Mitomycin was employed as positive control. Of the tested compounds, compounds **1**–**3** showed moderate cytotoxicity, with IC_50_ values ranging from 25 to 63 μM, and compounds **4**–**11** were found to be inactive, with IC_50_ values >100 μM (Table 3). The most cytotoxic compound is cytochalasin E (**1**). Cytochalasin-type compounds have been reported as promising lead compounds for anticancer drug discovery that reduce the proliferation of different cancer cell lines such as P388, A549 and KB cells [10,11]. α-Pyrone natural products are widespread in Nature and have a broad spectrum of biological activities [13]. The literature [14,15,16] has suggested that structural variations of the side chains attached to α*-*pyrones ring may make significant differences to the cytotoxicity, which is worth further investigation.

**Table 3:** Cytotoxicities of compounds **1**–**11** against two cancer cell lines ^a^.

	1	2	3	4	5	6	7	8	9	10	11
HepG2	25	59	45	>100	>100	>100	>100	>100	>100	>100	>100
Caski	29	63	53	>100	>100	>100	>100	>100	>100	>100	>100

^a^ Results are expressed as IC_50_ values in μM. An average value of three independent experiments is reported. Hep-G2 and Caski are human liver cancer cell line and human cervical cancer cell line, respectively.

## 3. Experimental Section

### 3.1. General Experimental Procedures

NMR spectra (^1^H at 400 MHz, ^13^C at 100 MHz) were measured on a Bruker Ultrashield TM Plus 400 MHz spectrometer (Faellanden, Switzerland) with tetramethylsilane as an internal standard and chemical shifts were recorded as δ values. EI and HREIMS spectra were recorded on DSQ II (Thermo Fisher Scientific Inc., Waltham, MA, USA) and MAT95XP (Thermo Electron Corp., Madison, WI, USA) mass spectrometers, respectively. Semipreparative HPLC was performed on a Dionex Ultimate 3000 system (Thermo Fisher Scientific Inc.) using a semipreparative C18 (COSMOSIL 5 μm, 10 mm × 250 mm) column coupled with an diode-array detector. TLC was performed on precoated silica gel GF_254_ (Merck, Darmstadt, Germany) plates (PTLC, Merck). Silica gel (200–300 mesh) for column chromatography was produced by Qingdao Marine Chemical Factory (Qingdao, China). Sephadex LH-20 was produced by Pharmacia Biotech (Uppsala, Sweden). UV spectra were taken on a UV-3100PC spectrometer (Hitachi, Ltd., Tokyo, Japan).

### 3.2. Isolation and Identification of the Strain

The endophytic fungus *Xylaria* sp. BM9 was isolated from a piece of fresh tissue from the inner part of a medicinal plant leaf of *Saccharum arundinaceum* Retz., collected from the Yichang (Hubei Province, China) in April 2011. The fungus was deposited as *Xylaria* sp. BM9 (GenBank accession numbers KC184129) at the Hubei Key Laboratory of Natural Products Research and Development, College of Chemistry and Life Sciences, China Three Gorges University, Yichang, China.

### 3.3. Seed and Mass Cultures of the Strain

The fungus *Xylaria* sp. BM9 was maintained on potato dextrose agar. Agar plugs containing the fungal strain were inoculated in 500 mL Erlenmeyer flasks, each containing 200 mL of potato dextrose broth. Flask cultures were incubated at 28 °C on a rotary shaker at 130 rpm for 3 days as seed culture.

Liquid fermentation was performed by the following procedure: each of the seed cultures (200 mL) was transferred into 500 mL Erlenmeyer flasks containing 200 mL of potato dextrose broth. These flasks were incubated at 28 °C on a rotary shaker at 130 rpm for 14 days. After fermentation, the culture (25 L) was centrifuged to yield the supernatant and a mycelial cake. The supernatant was extracted three times with an equal volume of EtOAc, the extracts were combined and solvent was removed under reduced pressure. The mycelial cake was immersed in 2 L of acetone and the organic layers were collected and removed under reduced pressure. Two residues were combined for purification.

Solid fermentation was carried out in 500 mL Erlenmeyer flasks containing 40 g rice, each flask was inoculated with the seed cultures (5.0 mL) and incubated at 25 °C for 30 days. The fermented material was soaked in EtOAc, and the organic solvent was evaporated to dryness under vacuum to afford the crude extract.

### 3.4. Extraction and Isolation of Compounds

The liquid fermentation residue (4.0 g) was chromatographed on a silica gel (200–300 mesh) column and eluted with petroleum ether–acetone (1:0, 9:1, 4:1, 2:1, 1:1, and 1:2, v/v) to yield six fractions (Fr. 1–Fr. 6). Fr. 3 (1.2 g) was further separated by semi-preparative reversed-phase HPLC on an ODS semi-preparative C18 column (COSMOSIL 5 μm, 10 mm × 250 mm) eluted with 30% MeCN/H_2_O to afford **6** (2.8 mg), **7** (5.4 mg), **8** (3.2 mg), **9** (1.5 mg), **10** (4.9 mg) and **11** (1.9 mg).

The solid fermentation residue (50 g) was chromatographed on a silica gel (200–300 mesh) column and eluted with petroleum ether–acetone (1:0, 9:1, 4:1, 2:1, 1:1, and 1:2, v/v) to yield nine fractions (Fr.1-Fr.9). Fr.2 (2.5 g) was further separated by chromatography on a Sephadex LH-20 column eluted with CHCl_3_–MeOH (v/v = 1/1) to yield seven fractions (Fr. 2.1–Fr. 2.7). Fr. 2.3 (200 mg) was further separated by semi-preparative reversed-phase HPLC on an ODS semi-preparative C18 column (COSMOSIL 5 μm, 10 mm × 250 mm) eluted with 75% MeCN/H_2_O to afford **3** (11.3 mg), **4** (12.9 mg) and **5** (14.6 mg). Fr. 2.5 (200 mg) was successively subjected to semi-preparative reversed-phase HPLC eluted with 85% MeCN/H_2_O to afford **2** (1.9 mg). The Fr. 4 (1.8 g) was purified by column chromatography over Sephadex LH-20 using an equal ratio mixture of methanol and chloroform as eluent to give **1** (40 mg).

*Cytochalasin E* (**1**): colorless needles; C_28_H_33_NO_7_; UV (MeOH) λ_max_ (logε) 224 (2.78), 258 (2.46) nm; ^13^C-NMR (acetone-*d*_6_): δ 212.9 (C-17), 170.3 (C-1), 150.1 (C-22), 142.4 (C-20), 137.4 (C-1'), 132.1 (C-14), 131.1 (C-2' & 6'), 129.5 (C-13), 129.1 (C-3' & 5'), 127.4 (C-4'), 121.7 (C-19), 87.8 (C-9), 77.6 (C-18), 61.2 (C-7), 57.7 (C-6), 53.6 (C-3), 47.5 (C-4), 47.0 (C-8), 44.5 (C-10), 41.1 (C-16), 40.0 (C-15), 36.9 (C-5), 25.0 (C-25), 20.4 (C-24), 19.6 (C-12), 13.1 (C-11); ESIMS *m/z* 518 [M+Na]^+^, 1013 [2M+Na]^+^.

*Cytochalasin K* (**2**): White amorphous solid; C_28_H_33_NO_7_; UV (MeOH) λ_max_ (logε) 224 (3.38), 258 (2.42) nm; ^13^C-NMR (CDCl_3_): δ 211.5 (C-17), 170.0 (C-1), 149.0 (C-22), 142.5 (C-20), 136.7 (C-1'), 133.6 (C-14), 131.8 (C-6), 129.4 (C-13), 129.3 (C-2' & 6'), 129.0 (C-3' & 5'), 127.2 (C-4'), 125.3 (C-5), 120.5 (C-19), 86.2 (C-9), 77.2 (C-18), 70.1 (C-7), 59.0 (C-3), 50.0 (C-8), 48.4 (C-4), 44.2 (C-10), 41.0 (C-16), 39.0 (C-15), 24.6 (C-25), 20.2 (C-24), 17.7 (C-11), 14.0 (C-12); ESIMS *m/z* 518 [M+Na]^+^, 1013 [2M+Na]^+^.

*Cytochalasin Z16* (**3**): White amorphous solid; C_28_H_33_NO_5_; UV (MeOH) λ_max_ (logε) 244 (3.73) nm; ^13^C-NMR (CDCl_3_): δ 206.1 (C-17), 171.3 (C-1), 170.0 (C-21), 149.1 (C-6), 143.7 (C-18), 138.1 (C-1'), 137.9 (C-14), 132.9 (C-19), 130.2 (C-2' & 6'), 129.8 (C-3' & 5'), 128.0 (C-4'), 126.6 (C-13), 115.8 (C-12), 84.1 (C-9), 69.9 (C-7), 54.4 (C-3), 50.8 (C-8), 49.9 (C-4), 44.6 (C-10), 40.6 (C-15), 40.3 (C-16), 37.2 (C-20), 32.7 (C-5), 18.5 (C-23), 15.0 (C-11), 13.5 (C-24); ESIMS *m/z* 486 [M+Na]^+^, 949 [2M+Na]^+^.

*Rosellichalasin* (**4**): White amorphous solid; C_28_H_33_NO_5_; UV (MeOH) λ_max_ (logε) 244 (3.75) nm; ^13^C-NMR (CDCl_3_): δ 205.4 (C-17), 171.6 (C-1), 169.0 (C-21), 143.0 (C-18), 136.5 (C-1'), 135.2 (C-14), 131.7 (C-19), 129.5 (C-2' & 6'), 128.8 (C-3' & 5'), 127.1 (C-4'), 125.6 (C-13), 84.5 (C-9), 60.1 (C-3), 57.1 (C-6), 53.8 (C-7), 48.9 (C-4), 46.9 (C-8), 44.1 (C-10), 39.7 (C-5), 39.6 (C-15), 36.4 (C-20), 35.9 (C-16), 19.5 (C-12), 17.3 (C-11), 12.8 (C-23), 12.7 (C-24); ESI-MS *m/z* 486 [M+Na]^+^, 949 [2M+Na]^+^.

*Cytochalasin Z17* (**5**): White amorphous solid; C_28_H_33_NO_5_; UV (MeOH) λ_max_ (logε) 242 (3.71) nm; ^13^C-NMR (CDCl_3_): δ 205.5 (C-17), 171.6 (C-1), 168.9 (C-21), 143.2 (C-18), 137.1 (C-14), 137.0 (C-1'), 132.9 (C-6), 131.5 (C-19), 129.2 (C-2' & 6'), 128.9 (C-3' & 5'), 127.1 (C-4'), 126.4 (C-13), 125.1 (C-5), 83.7 (C-9), 69.6 (C-7), 59.2 (C-3), 50.0 (C-8), 49.6 (C-4), 43.7 (C-10), 40.1 (C-16), 39.7 (C-15), 37.4 (C-20), 17.5 (C-11), 17.2 (C-23), 14.1 (C-12), 13.0 (C-24); ESI-MS *m/z* 486 [M+Na]^+^, 949 [2M+Na]^+^.

*Xylapyrone A* (**6**): white amorphous powder; UV (MeOH) λ_max_ (logε) 201 (0.16), 280 (2.94) nm; ^1^H-NMR (DMSO-*d*_6_) and ^13^C-NMR (DMSO-*d*_6_) spectral data see Table 1. HREIMS *m/z* 254.1147 [M]^+^ (calcd for C_13_H_18_O_5_, 254.1154).

*Xylapyrone B* (**7**): white amorphous powder; UV (MeOH) λ_max_ (logε) 201 (0.14), 280 (2.93) nm; ^1^H-NMR (DMSO-*d*_6_) and ^13^C-NMR (DMSO-*d*_6_) spectral data see Table 1. HREIMS *m/z* 256.1308 [M]^+^ (calcd for C_13_H_20_O_5_, 256.1311).

*Xylapyrone C* (**8**): white amorphous powder; UV (MeOH) λ_max_ (logε) 201 (0.15), 280 (2.93) nm; ^1^H-NMR (DMSO-*d*_6_) and ^13^C-NMR (DMSO-*d*_6_) spectral data see Table 1. HREIMS *m/z* 212.1045 [M]^+^ (calcd forC_11_H_16_O_4_, 212.1049).

*Xylapyrone D* (**9**): white amorphous solid; UV (MeOH) λ_max_ (logε) 201 (0.14), 280 (2.98) nm; ^1^H-NMR (DMSO-*d*_6_) and ^13^C-NMR (DMSO-*d*_6_) spectral data see Table 2. HREIMS *m/z* 228.0991 [M]^+^ (calcd for C_11_H_16_O_5_, 228.0998).

*Xylapyrone E* (**10**): pale yellow oil; UV (MeOH) λ_max_ (logε) 201 (0.15), 280 (2.99) nm; ^1^H-NMR (DMSO-*d*_6_) and ^13^C-NMR (DMSO-*d*_6_) spectral data see Table 2. HREIMS *m/z* 198.0886 [M]^+^ (calcd for C_10_H_14_O_4_, 198.0892).

*Xylapyrone F* (**11**): white amorphous powder; UV (MeOH) λ_max_ (logε) 201 (0.17), 280 (2.99) nm; ^1^H-NMR (DMSO-*d*_6_) and ^13^C-NMR (DMSO-*d*_6_) spectral data see Table 2. HREIMS *m/z* 184.0729 [M]^+^ (calcd for C_9_H_12_O_4_, 184.0736).

### 3.5. Cytotoxicity Test

The cancer cell lines CaSki and HepG2 were obtained from the Shanghai Institute of Cell Biology, Chinese Academy of Science (Shanghai, China). All cells were maintained in RPMI-1640 medium supplemented with 10% fetal calf serum, 25 mM HEPES buffer, 2 mmol/L l-glutamine, 100 µg/mL streptomycin, and 100 U/mL penicillin. Cultures were incubated in a humidified atmosphere of 5% CO_2_ at 37 °C. Cells (1 × 10^4^/well) were seeded in supplemented culture medium (100 μL/well) in a 96-well plate and incubated for 24 h. The medium was then replaced with a test compound-containing medium, and the cells were further incubated for 48 h. All experiments were run in parallel with controls (0.1% DMSO without test compounds) and the cell viabilities were evaluated by MTT assays. The absorbance of formazan formed was measured at 570 nm by a microplate reader. The concentrations resulting in 50% inhibition of cell proliferation/survival (IC_50_) as measured by this assay are given in Table 3. Each experiment was repeated three times.

## 4. Conclusions

The *Xylaria* genus is an ubiquitous filamentous fungus, often isolated from marine environments and terrestrial sources, that can produce various types of secondary metabolites, including typical cytochalasins [17], terpenoids [18], benzofurans [19], xanthones [20], and cyclopeptides [21]. Long-chain α-pyrones are widespread in Nature and most of the PKS genes have been involved in the biosynthesis of their derivatives from acetate units [22,23]. A few papers have reported α-pyrone metabolites from *Xylaria* sp. Pukachaisirikul *et al.* described the isolation of one known α-pyrone derivative [24]. In this paper, five known cytochalasin-type analogues **1**–**5** were isolated as the major metabolites from a solid rice medium culture of *Xylaria* sp. BM9. However, applying liquid culture conditions to *Xylaria* sp. BM9 resulted in the identification of six new α-pyrone derivatives, xylapyrones A–F (compounds **6**–**11**), with different biogenetic origin, in which its PKS genes for the biosynthesis of complex liquids were actived. Xylapyrones A–F were evaluated for *in vitro* cytotoxicity against two cancer cell lines (Hep-G2 and Caski) and found to be inactive. Further bioassay evaluation of the compounds against fungi and other targets are ongoing, so it is an issue that we don’t have sufficient amounts of these compounds to determine the absolute stereochemistry of compounds **6**, **7** and **9**.

## Supplementary Materials

Supplementary materials can be accessed at: http://www.mdpi.com/1420-3049/20/05/7940/s1.
