# Supplementary material for: Effect of Culture Conditions on Metabolite Production of Xylaria sp
Source: Molecules. 2015 Apr 30;20(5):7940–50. doi: 10.3390/molecules20057940 (PMC6272309; doi:10.3390/molecules20057940)
Supplement: Supplementary file 1 [file molecules-20-07940-s001.pdf]

Supplementary Materials

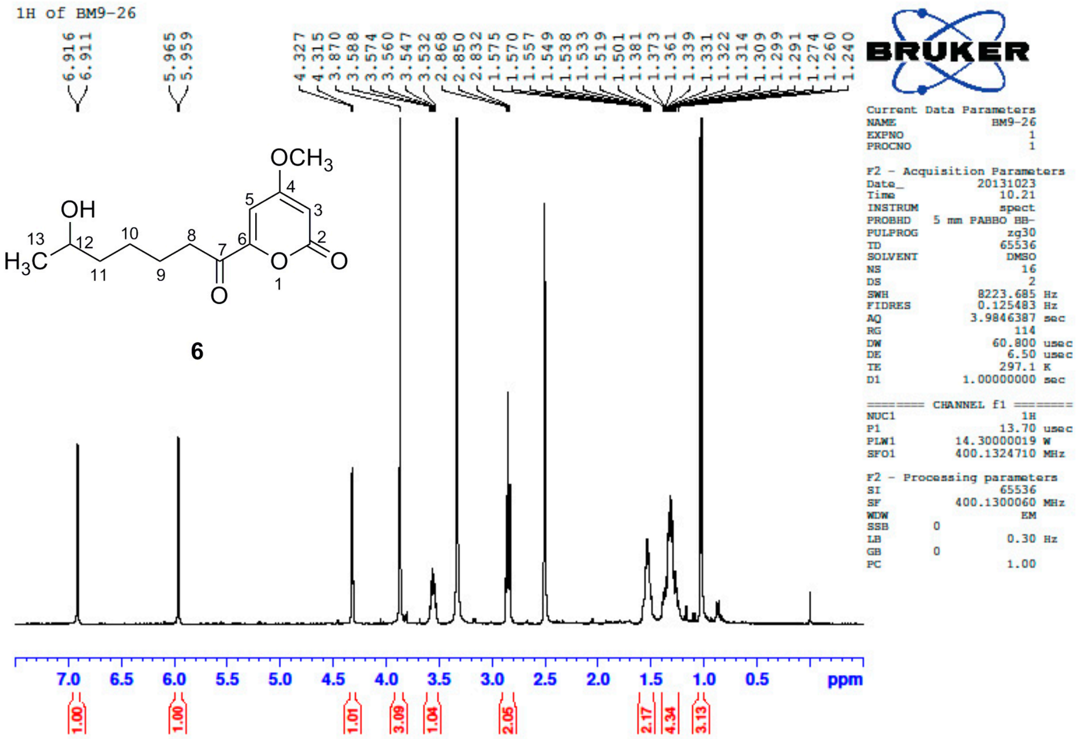

Figure S1. ^{1}H-NMR spectrum of xylapyrones A (**6**) (400MHz, DMSO).

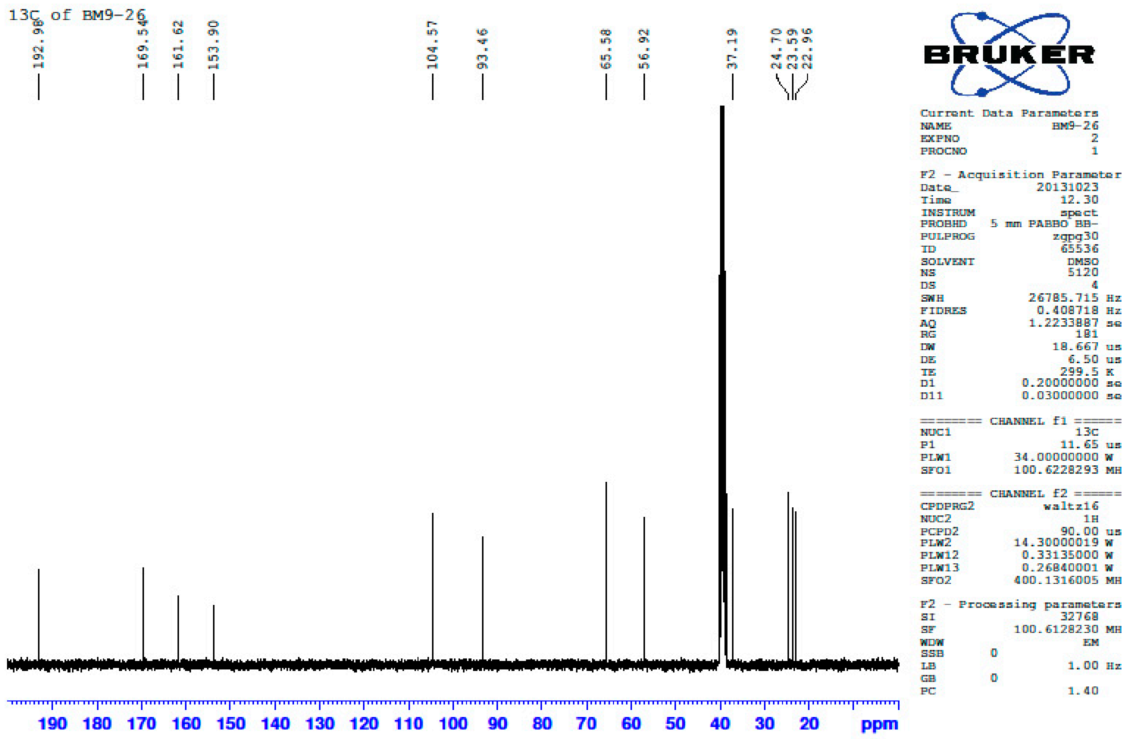

Figure S2. ^{13}C-NMR spectrum of xylapyrones A (**6**) (100MHz, DMSO).

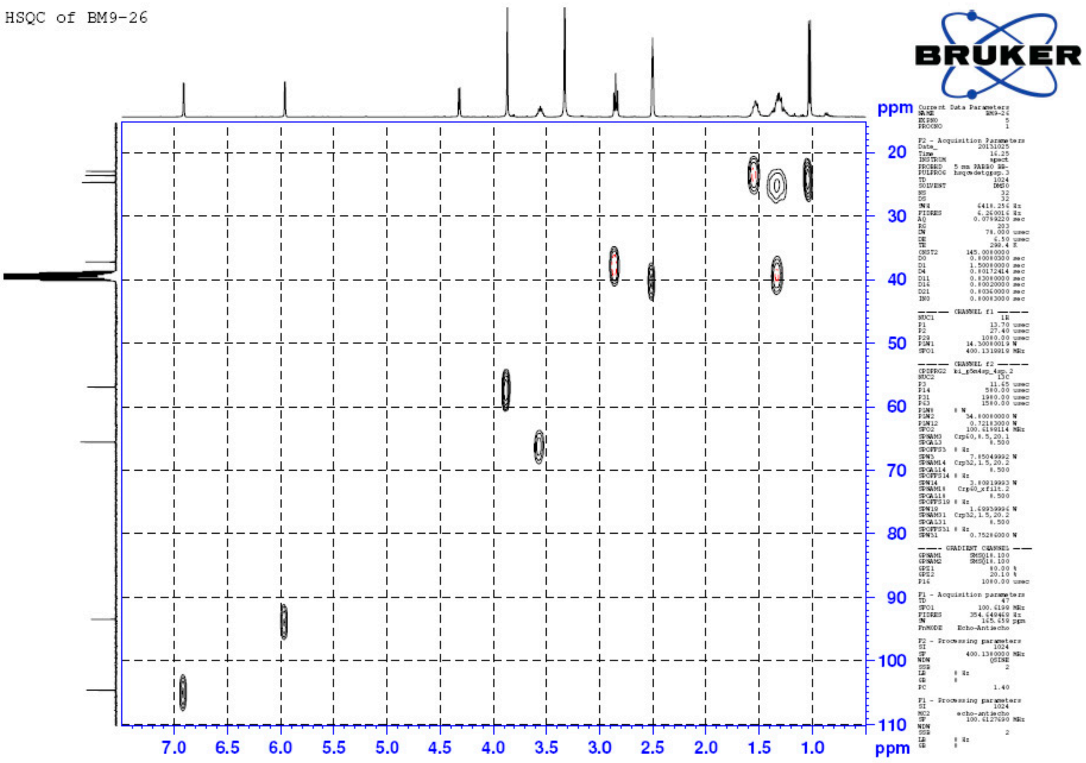

Figure S3. HSQC spectrum of xylapyrones A (**6**).

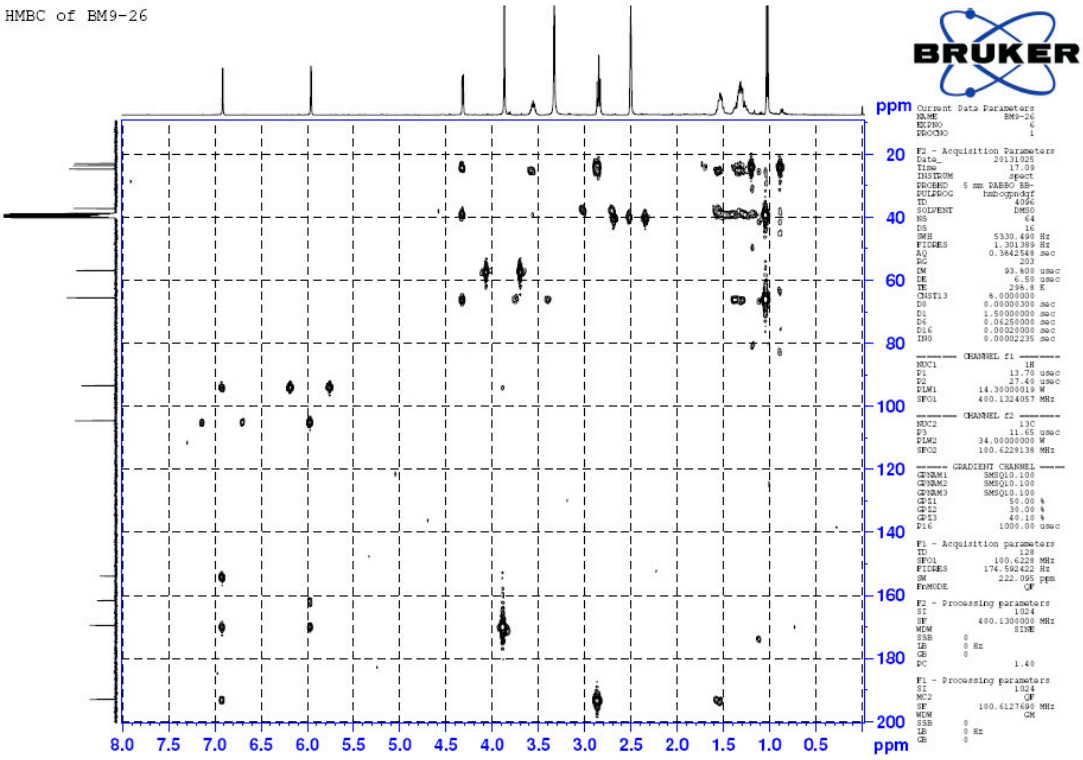

Figure S4. HMBC spectrum of xylapyrones A (**6**).

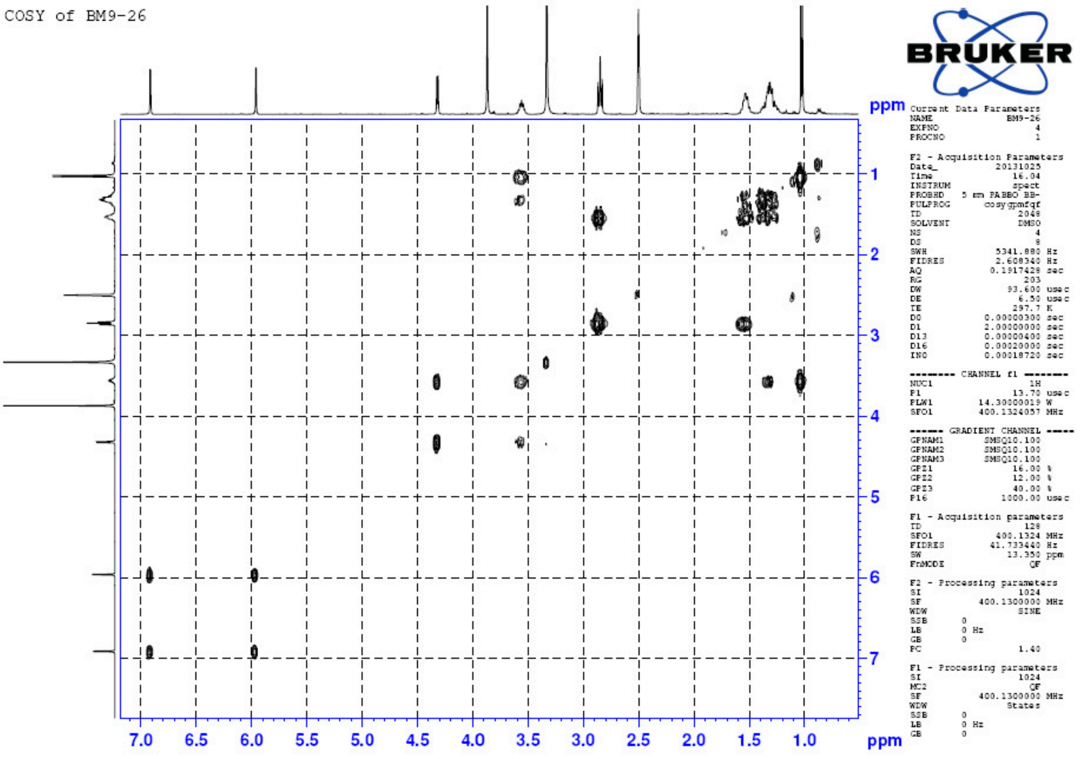

Figure S5. COSY spectrum of xylapyronesA (**6**).

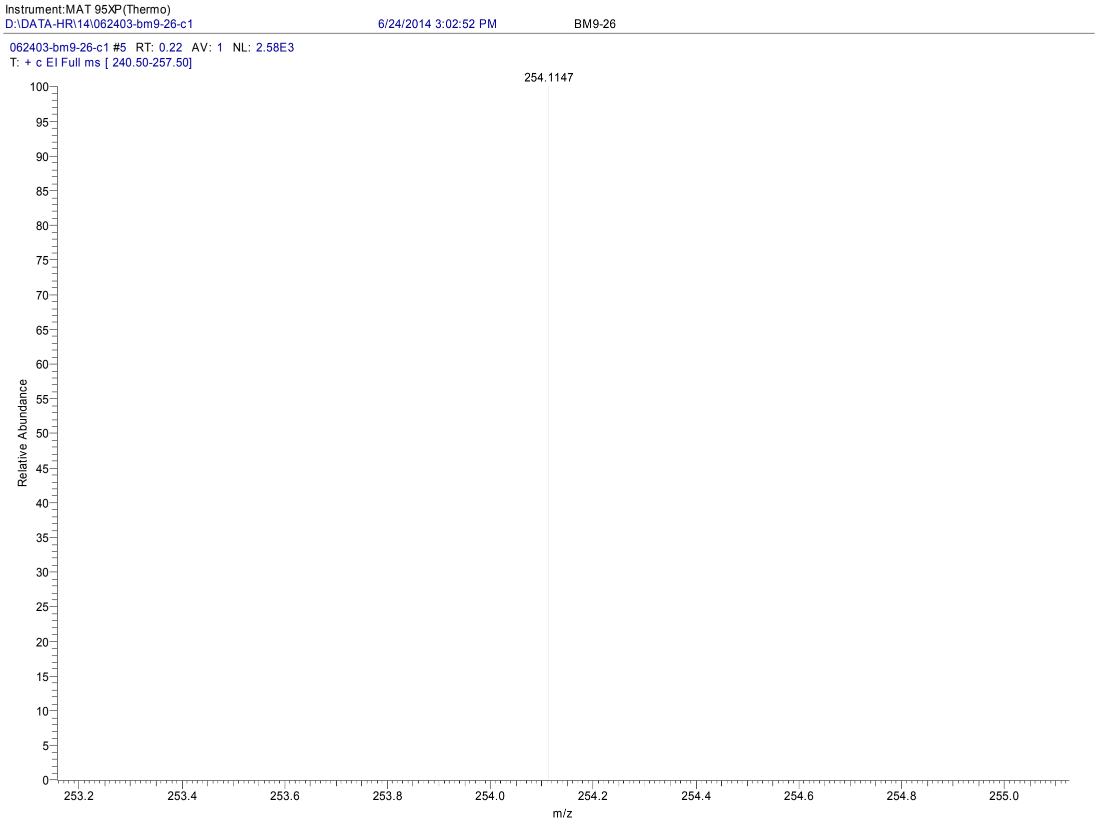

Figure S6. HREIMS spectrum of xylapyrones A (**6**).

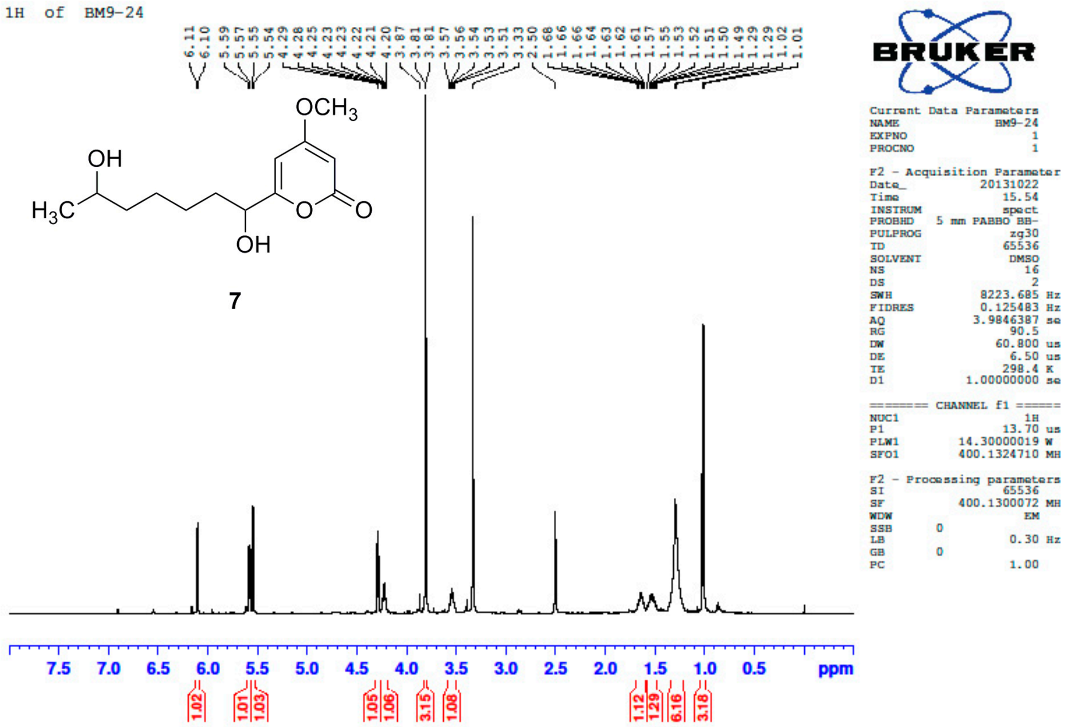

Figure S7. ^{1}H-NMR spectrum of xylapyrones B (**7**) (400MHz, DMSO).

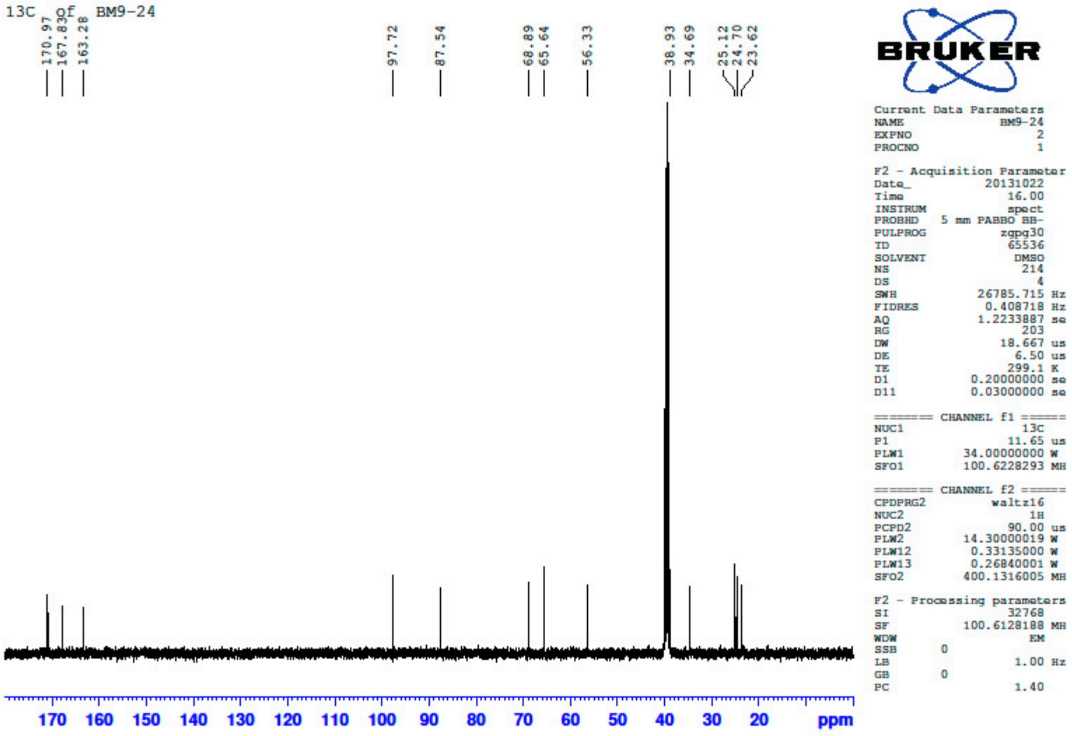

Figure S8. ^{13}C-NMR spectrum of xylapyrones B (**7**) (100MHz, DMSO).

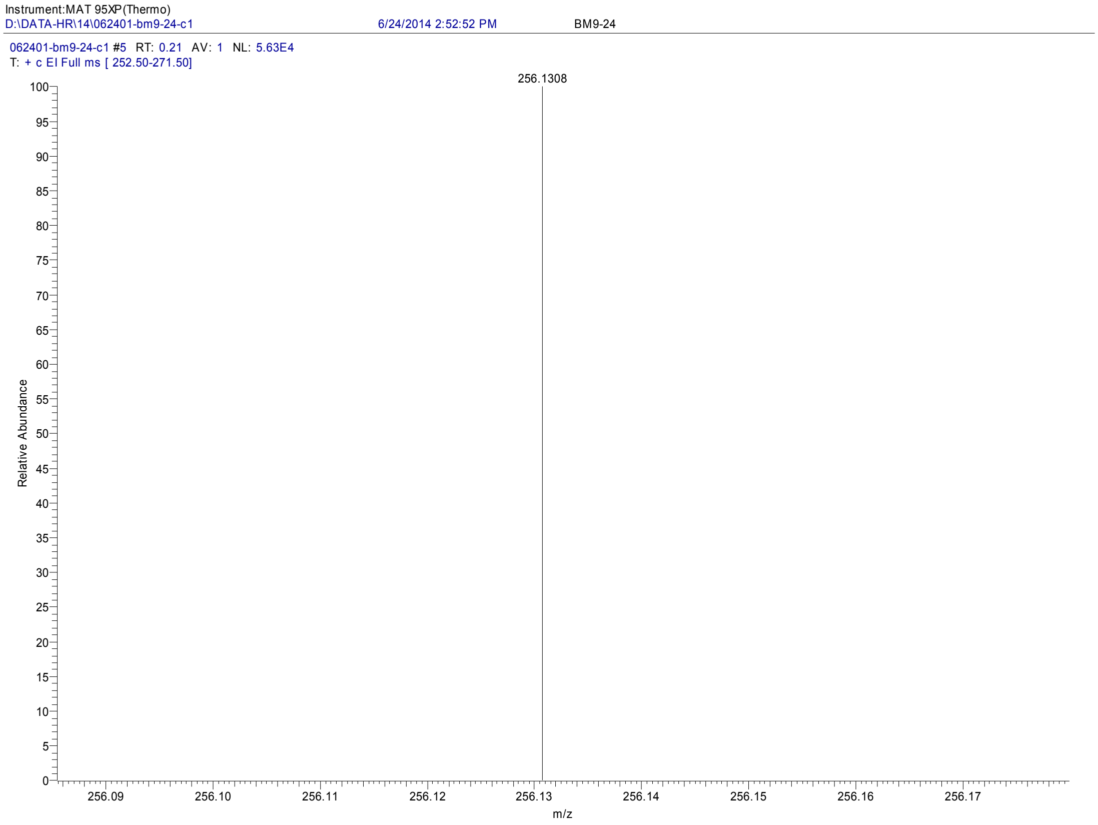

Figure S9. HREIMS spectrum of xylapyrones B (**7**).

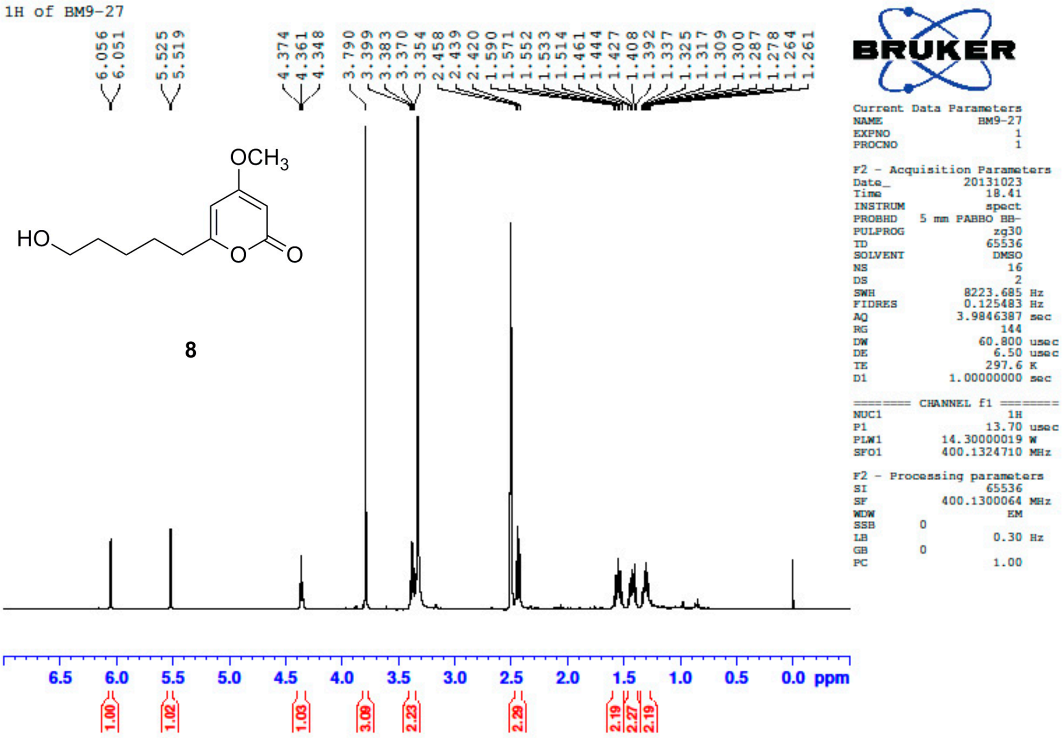

Figure S10. ^{1}H-NMR spectrum of xylapyrones C (**8**) (400MHz, DMSO).

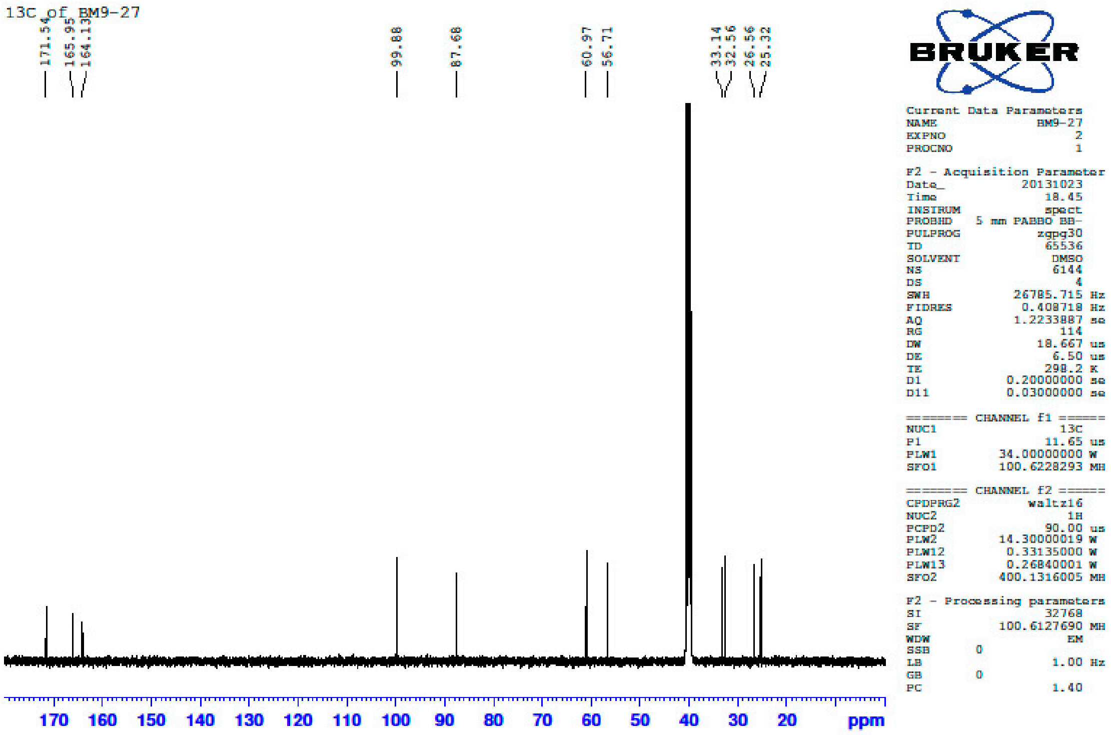

Figure S11. ^{13}C-NMR spectrum of xylapyrones C (**8**) (100MHz, DMSO).

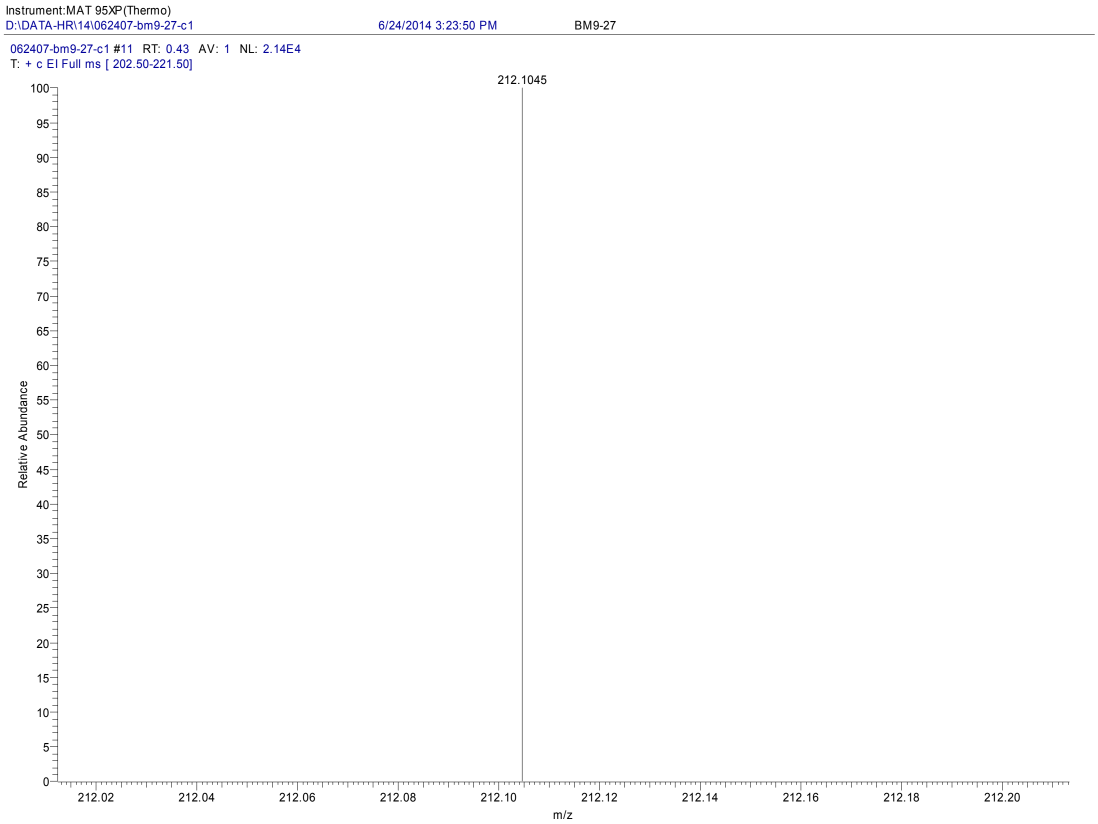

Figure S12. HREIMS spectrum of xylapyrones C (**8**).

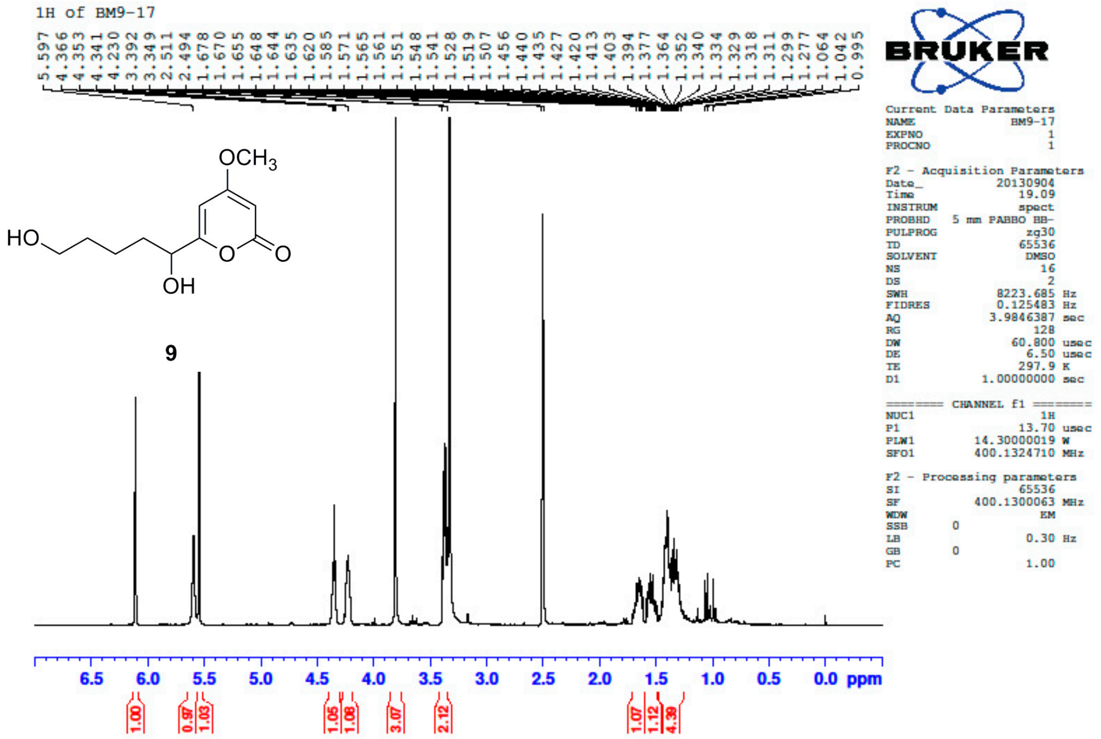

Figure S13. ^{1}H-NMR spectrum of xylapyrones D (**9**) (400MHz, DMSO).

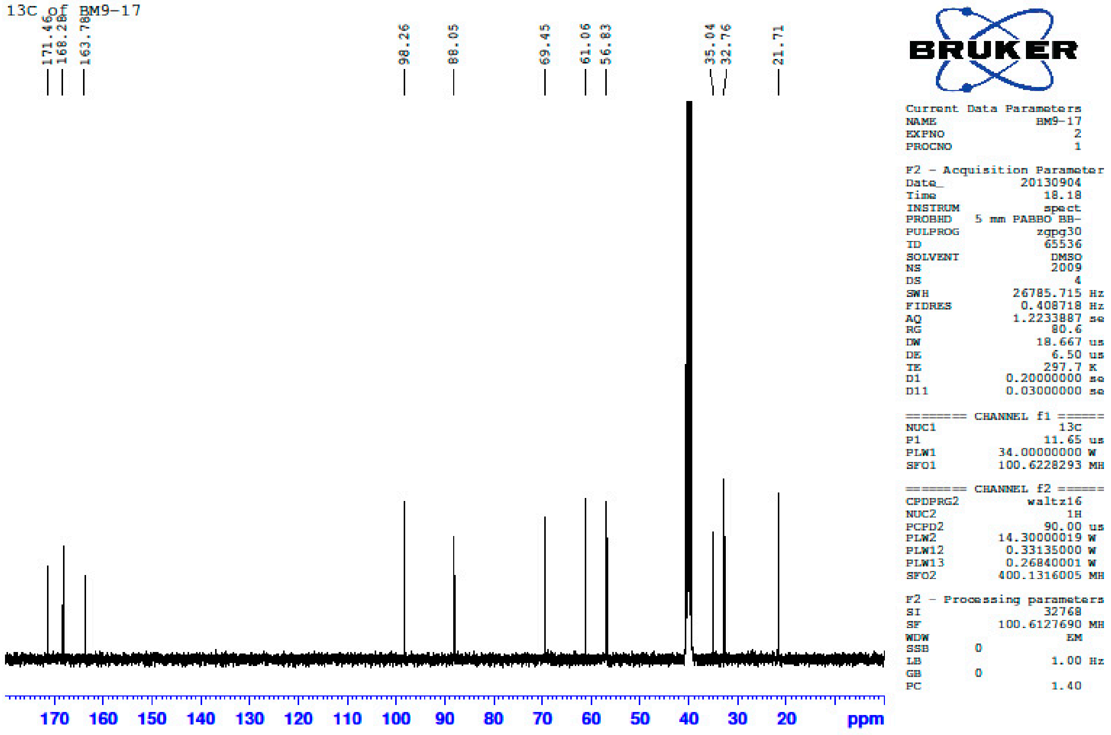

Figure S14. ^{13}C-NMR spectrum of xylapyrones D (**9**) (100MHz, DMSO).

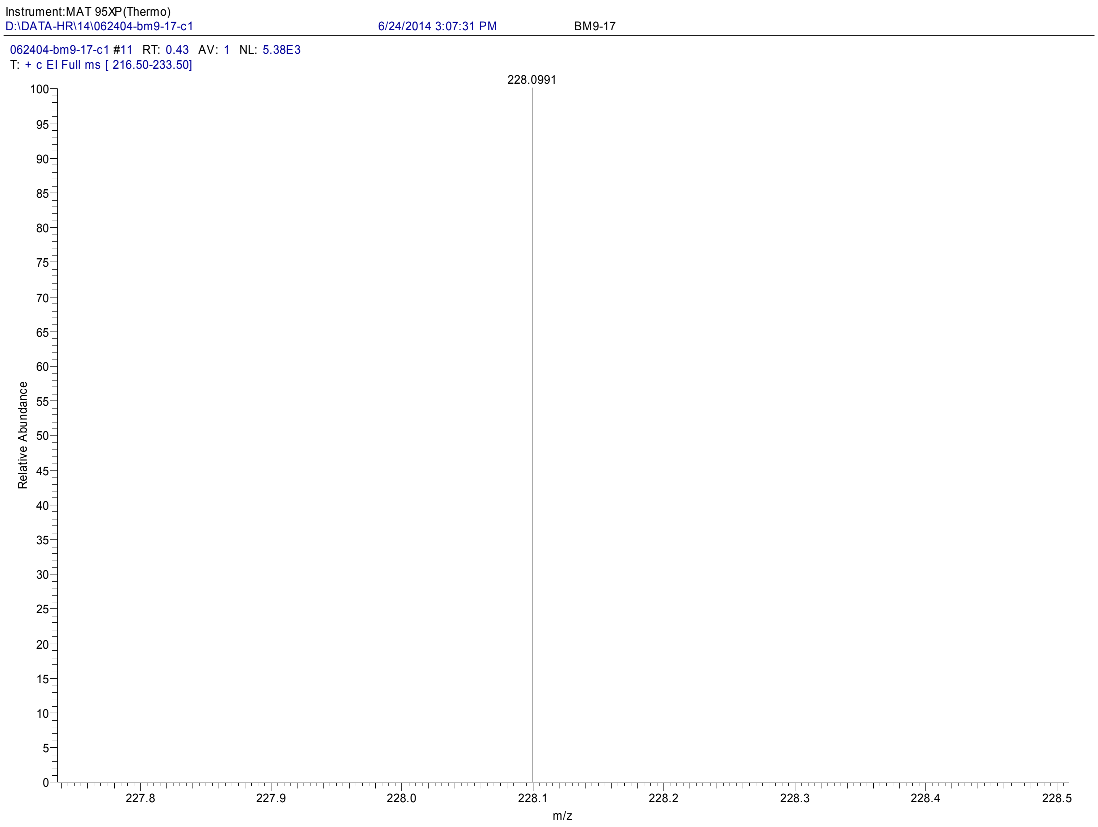

Figure S15. HREIMS spectrum of xylapyrones D (**9**).

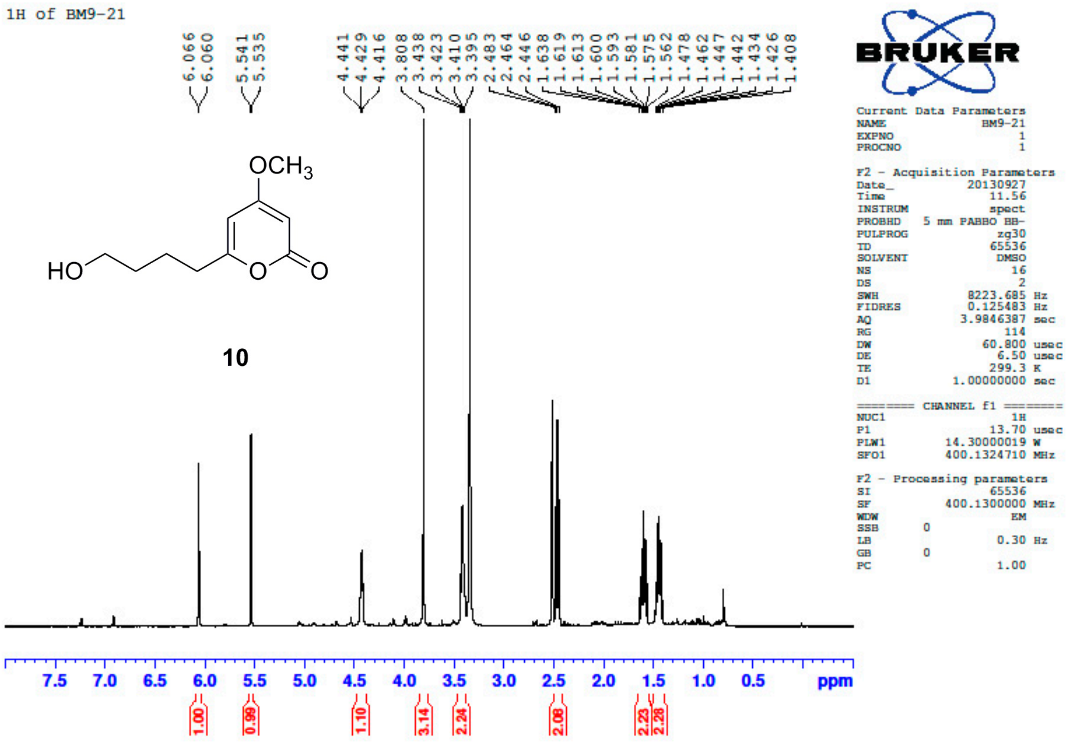

Figure S16. ^{1}H-NMR spectrum of xylapyrones E (**10**) (400MHz, DMSO).

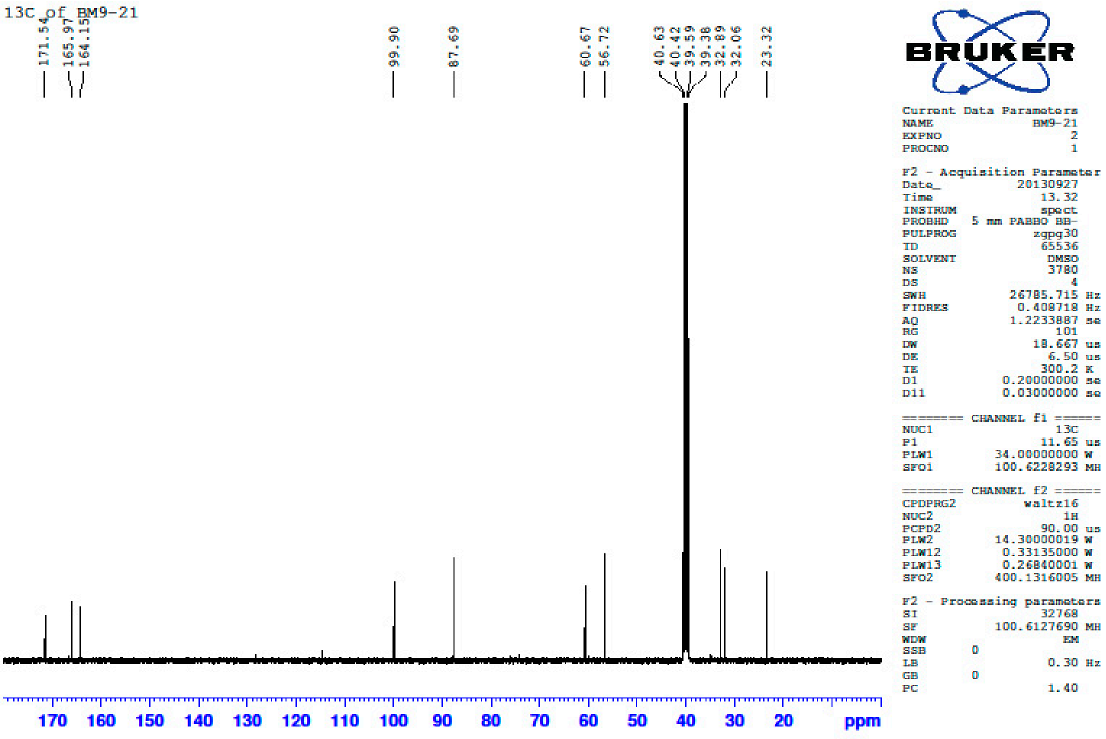

Figure S17. ^{13}C-NMR spectrum of xylapyrones E (**10**) (100MHz, DMSO).

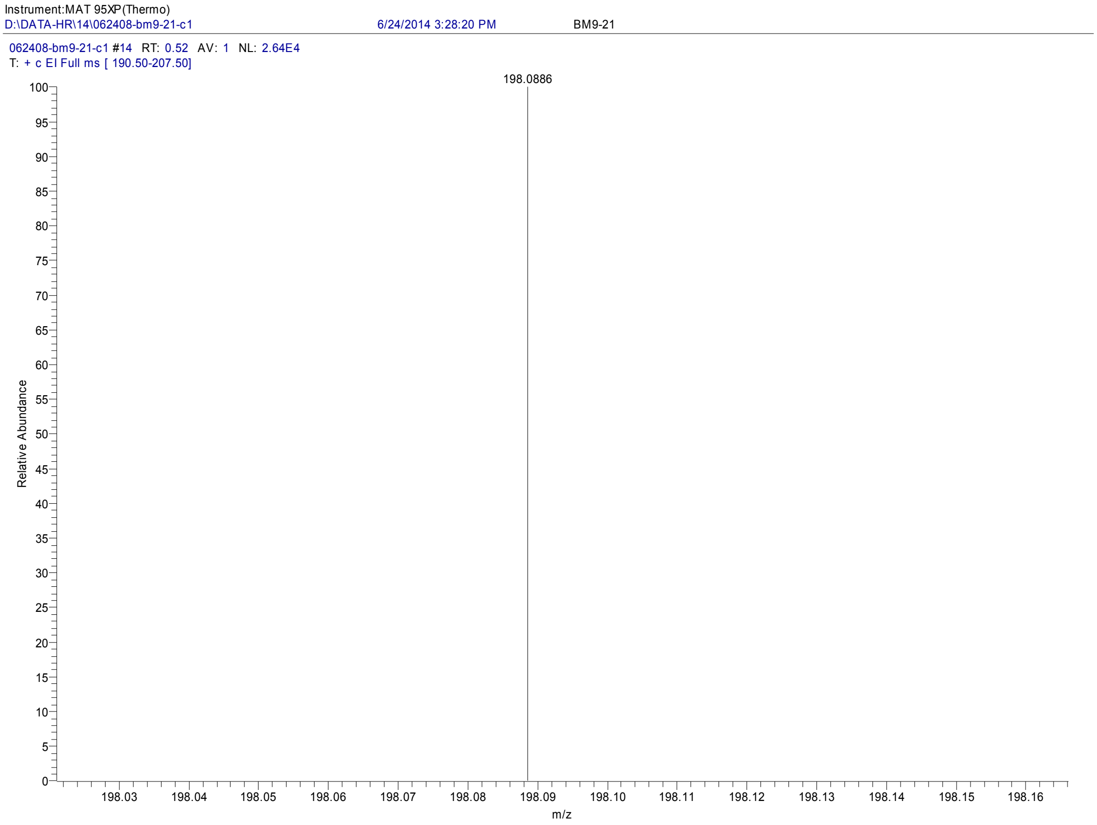

Figure S18. HREIMS spectrum of xylapyrones E (**10**).

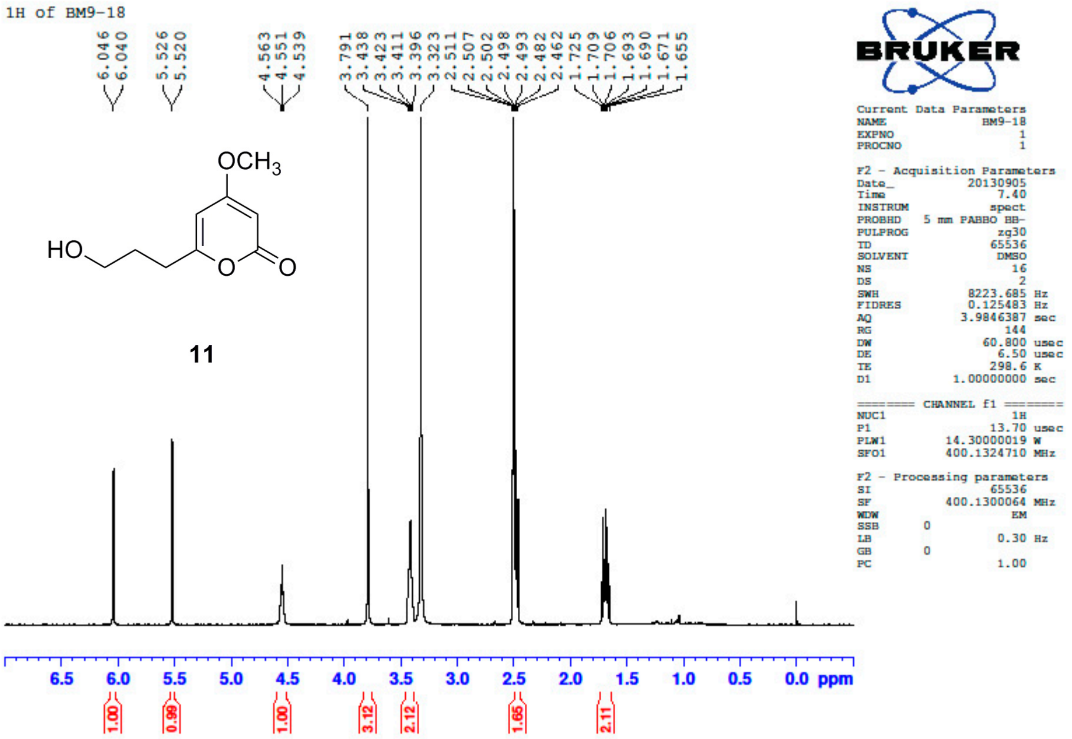

Figure S19. ^{1}H-NMR spectrum of xylapyrones F (**11**) (400MHz, DMSO).

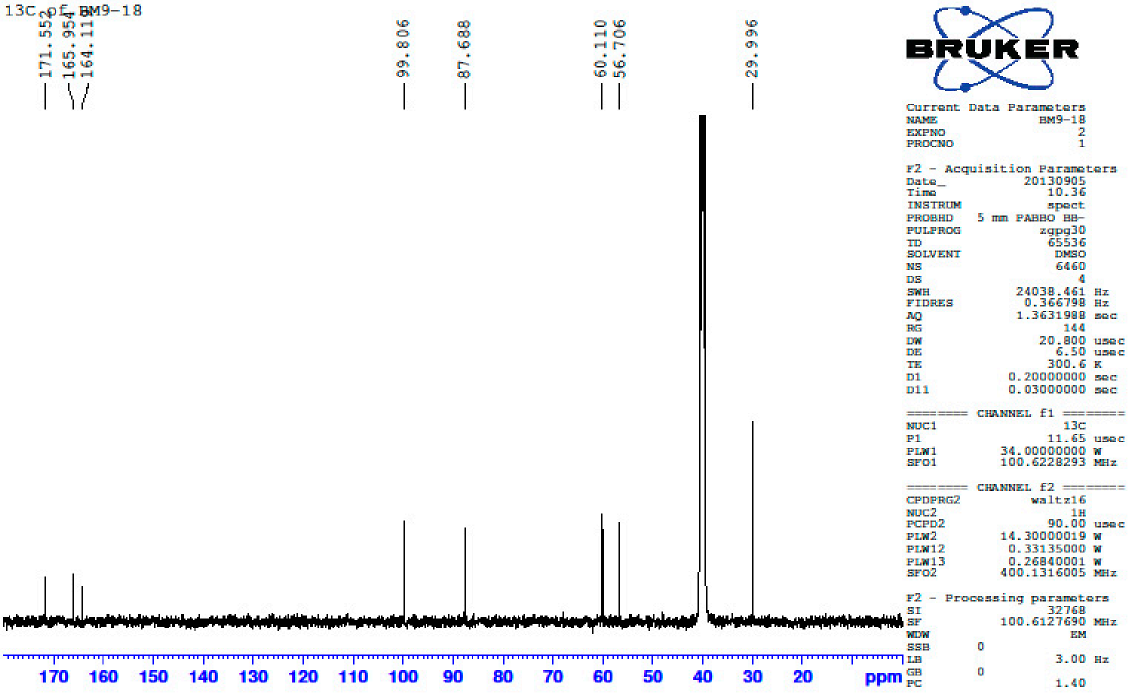

Figure S20. ^{13}C-NMR spectrum of xylapyrones F (**11**) (100MHz, DMSO).

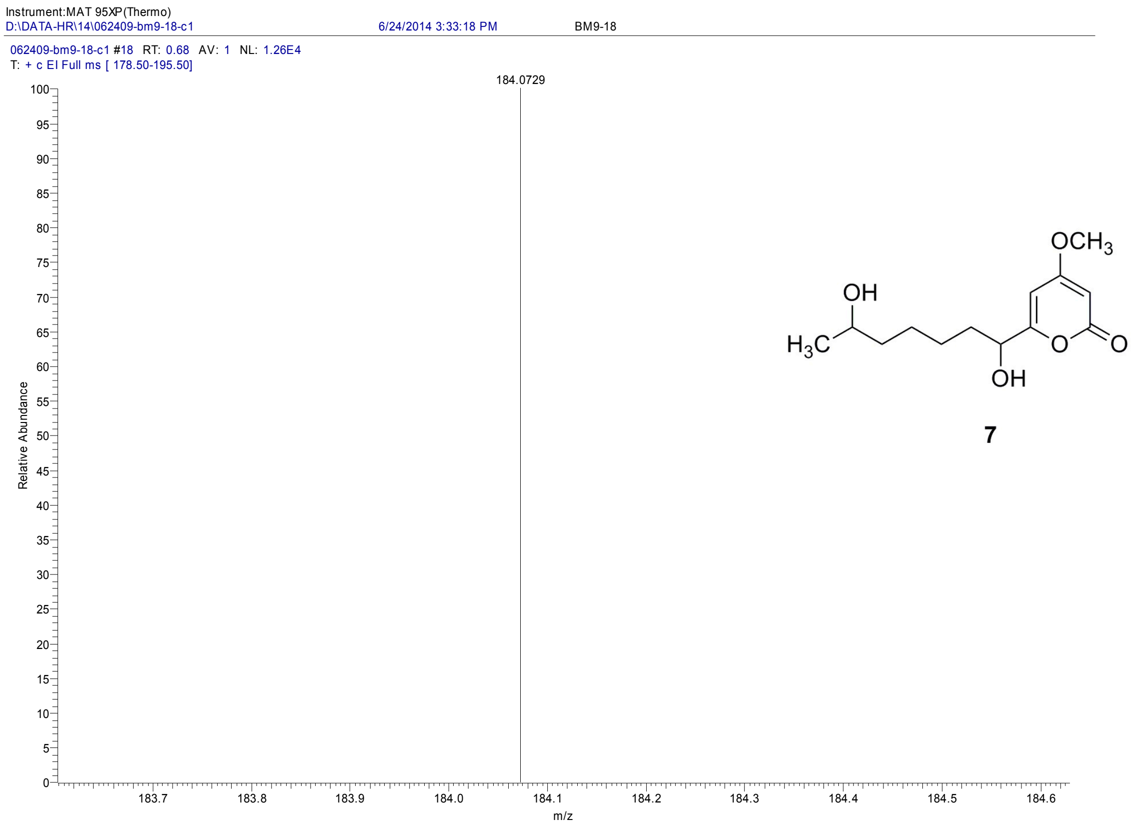

7

Figure S21. HREIMS spectrum of xylapyrones F (**11**).
